# Special Issue “New Updates in Oral and Maxillofacial Surgery”

**DOI:** 10.3390/jpm14070705

**Published:** 2024-07-01

**Authors:** Fabio Maglitto, Chiara Copelli, Alfonso Manfuso, Stefan Cocis, Giovanni Salzano

**Affiliations:** 1 Maxillo-Facial Surgery, Interdisciplinary Department of Medicine, University of Bari, 70100 Bari, Italy; chiara.copelli@uniba.it (C.C.); alfonso.manfuso@policlinic.ba.it (A.M.); stefan.cocis@policlinico.ba.it (S.C.); 2 Maxillofacial Surgery Unit, Department of Neurosciences, Reproductive and Odontostomatological Sciences, University of Naples Federico II, 80131 Naples, Italy; giovannisalzanomd@gmail.com

## 1. Introduction

In the ever-evolving landscape of medical science, few fields have witnessed as profound a transformation as oral and maxillofacial surgery. With each passing year, the integration of cutting-edge technology, innovative techniques, and personalized approaches is redefining the boundaries of what is possible in the realm of maxillofacial and head and neck reconstruction. The advent of digital imaging, precision planning, and minimally invasive procedures has ushered in a new era of patient-centered care and enhanced treatment outcomes.

The Special Issue titled “New Updates in Oral and Maxillofacial Surgery” serves as a guide to the dynamic nature of this discipline. By delving into the latest developments and future trends, it encapsulates the spirit of progress that defines oral and maxillofacial surgery today.

At the forefront of this paradigm shift is the emphasis on personalized treatment modalities. From digital impressions to intricate sterolithographic modeling, the ability to tailor interventions to the unique anatomical and physiological characteristics of each patient has become paramount. This personalized approach not only enhances precision, but also minimizes the risk of complications, ensuring optimal results and patient satisfaction.

The advent of AI can completely change the paradigms of medicine. Chat-Based Generative Pre-trained Transformer (ChatGPT) is an advanced artificial intelligence (AI) language model developed by Open Artificial Intelligence. With regard to head and neck disciplines, Vaira et al. [1] demonstrated AI’s ability to resolve complex clinical scenarios, but it still falls short of being considered a reliable support for the decision-making process of specialists in head–neck surgery.

Central to the digital revolution is the emergence of digital impressions and 3D modeling. Traditionally, the process of obtaining dental impressions was laborious and uncomfortable for patients. However, with the advent of intraoral scanners [2], we now offer our patients a more comfortable and efficient alternative. These digital impressions not only streamline the treatment process, but also enable us to achieve unprecedented accuracy in the fabrication of prosthetic restorations and orthognathic surgical planning.

Moreover, 3D planning has emerged as a cornerstone of contemporary oral and maxillofacial surgery. Through sophisticated software algorithms and computer-aided design (CAD) technologies [3,4,5], we can meticulously plan complex procedures, such as orthognathic surgery and implant placement, with precision and predictability. This level of preoperative planning not only optimizes surgical outcomes, but also minimizes intraoperative complications and reduces patient morbidity.

As discussed by Tian et al. [6] in their review, 3D printing is often used for digital imaging in surgical planning, custom surgical devices, and patient–physician communication.

In parallel, minimally invasive surgery has gained prominence as a preferred approach in the management of craniofacial pathologies. Endoscopic and functional surgery techniques have revolutionized our ability to access and treat complex anatomical regions with minimal disruption to surrounding tissues. These approaches not only result in faster recovery times and reduced postoperative pain, but also preserve aesthetic and functional outcomes for our patients. As demonstrated by Bartholomew et al. [7], the combinations between 3D endoscopy and surgical navigation has the potential to improve surgical efficiency, economy of motion, and safety for the patient.

As we navigate the landscape of oral and maxillofacial surgery, we remain steadfast in our commitment to addressing a diverse array of pathologies, including head and neck surgery, oral cancer, and reconstructive surgery. Augmented reality represents the most innovative approach to translate virtual planning for real patients, as it merges the digital world with the surgical field in real time. Surgeons can access patient-specific data directly within their field of view, through dedicated visors. In head and neck surgical oncology, augmented reality systems overlay critical anatomical information onto the surgeon’s visual field [8,9].

In particular, the management of oral cancer poses a significant clinical challenge, demanding a multidisciplinary approach and innovative diagnostic and therapeutic strategies [10,11]. Through collaborative efforts and cutting-edge research, we strive to improve early detection, enhance treatment efficacy, and optimize long-term outcomes for patients afflicted by this devastating disease.

Furthermore, reconstructive surgery continues to play a pivotal role in restoring form and function following trauma, congenital anomalies, or oncologic resections. As discussed by Garajei et al., the virtual surgical planning technique resulted in better facial symmetry and superior esthetic outcomes compared with the conventional technique [12]. Advances in biomaterials, tissue engineering, and microsurgical techniques have expanded the repertoire of reconstructive options available to us, enabling us to achieve remarkable outcomes and improve quality of life for our patients.

## 2. An Overview of Published Articles

In Andreas Sakkas’s paper [13], the author explores the prevalence of challenging airway situations and urgent tracheostomy procedures in individuals with orofacial infections arising from the mandible, as well as methods for identifying factors that may predict difficult intubation. The frequency of challenging airway situations related to breathing, visualizing the larynx, and inserting a breathing tube was studied using descriptive methods. The relationships between possible factors that could affect difficult intubation were investigated through multivariable analysis. The highest occurrence of challenging intubation occurred in patients with infections in the masseteric–mandibular space (42.6%), then in infections of the floor of the mouth (40%), and, finally, in infections in the pterygomandibular space (23.5%). Breathing difficulties and noisy breathing were not linked to the site of infection. The researchers concluded that there was a high prevalence of difficult airway situations in patients with orofacial infections originating in the mandible. Advanced age, restricted mouth opening, a higher Mallampati classification, and a higher Cormack–Lehane grade were identified as dependable indicators of challenging intubation.

The study conducted by Gonzalez-Perez and colleagues [14] examines the use of total joint replacement for immediate reconstruction following ablative surgery for primary tumors affecting the temporo-mandibular joint. In their case series, the researchers emphasized the effectiveness of surgically placing TMJ prostheses in significantly reducing pain levels and TMJ dysfunction resulting from tumor-related TMJ pathology. This retrospective analysis primarily targeted cases where the TMJ was extensively damaged due to various tumors. Compared to alternative reconstructive procedures like costochondral or sternoclavicular grafts, utilizing a TMJ prosthesis can shorten surgery duration and hospital stay while offering immediate functionality without donor site morbidity. TMJ replacement is typically viewed as a final option in the surgical management of TMJ disorder and is considered the preferred approach for immediate reconstruction post-ablative surgery for primary TMJ tumors.

The third article by Boschetti et al. [15] examines the effectiveness of using fat grafts in parotidectomy surgeries. The patients in the study all underwent partial or complete parotidectomy with autologous en bloc dermal fat graft reconstruction. Pre- and post-operative contrast-enhanced MRI scans were performed on all patients. Positive feedback from patients on the cosmetic results, along with validation from the radiology team, supports fat grafting as a successful and safe surgical procedure, even in cases with malignant lesions. The authors conclude that fat grafting is highly successful in terms of aesthetics, procedure duration, and safety during oncological follow-up, consolidating the role of a key technique in craniofacial reconstructive surgery.

The fourth article in this Special Issue, authored by Ho et al. [16], presents a preliminary examination of the impact of surgical precision in maxillomandibular advancement (MMA) on obstructive sleep apnea (OSA) patients. The study specifically looks at how advancements in the maxilla and mandible, as well as counter-clockwise rotation, relate to the reduction in the relative apnea hypopnea index (AHI). This research highlights the significance of recognizing surgical inaccuracies in MMA procedures for OSA patients and emphasizes the importance of increased awareness among surgeons and future research efforts.

The article by Antunez-Conde Hidalgo [17] compared the conventional technique and the customized guided surgery for genioplasty. Genioplasty is a common surgical procedure in the field of maxillofacial surgery. Improvements in facial reconstructive surgery have led to lower risk and more consistent outcomes. This study compares traditional genioplasty with a new surgical technique based on virtual surgical planning, CAD-CAM cutting guides, and custom-made plates for patients.

Serree and colleagues [18] conducted a study to evaluate the precision of a novel MD (PirifixTM) for assisting surgeons in positioning the upper dental arch (UDA) during Le Fort I osteotomies (LFIOs). The PirifixTM, developed by Ennoïa in Besançon, France, is a bone-supported device designed to conform to the lower part of the piriform orifice’s anatomy. It serves as an alternative to existing methods for UDA positioning in LFIO procedures. This initial investigation was performed on a 3D-printed model without soft tissue to demonstrate the efficacy of the PirifixTM. However, further research, incorporating facial soft tissue and input from other surgeons, is necessary to validate its effectiveness. PirifixTM could potentially have a significant impact on the performance of complex movements that involve multiple rotations and translations. Further investigation into the use of PirifixTM for complex movements is warranted to fully understand its potential benefits and applications.

Lee’s research [19] delves into the nasal dimension using Cone-Beam Computed Tomography. The morphology of the nasal cavity is crucial not only in clinical settings, but also in forensic science. However, previous studies have mainly focused on comparing sexes, so it is important to explore how nasal dimensions correspond to facial and nose dimensions. The authors want to determine whether nasal cavity size varies based on sex, facial index (FI), and nasal index (NI). Lee and colleagues examined CBCT data from 100 patients at Dankook University’s dental hospital. Their results indicated that nasal cavity sizes did indeed differ according to sex, FI, and NI. These findings have implications for personalized surgeries in clinical practice and future studies on nasal cavity anatomy.

In their study, Onica et al. [20] share their clinical findings regarding a subperiosteal jaw implant as an alternative for patients with insufficient bone height, who cannot undergo traditional endosseous implants without extensive bone grafting or augmentation procedures. The success of these implants largely depends on the biotype of the gingiva, which influences the quality and quantity of the surrounding soft tissues. The objective of this research is to discuss a newly designed subperiosteal jaw implant through their 6-year clinical experience.

The findings reveal that subperiosteal implant-supported hybrid prostheses, created using digital planning and guided surgery, have yielded disappointing outcomes with only a 25% success rate after 6 years.

## 3. Conclusions

This collection of articles dedicated to the new updates in oral and maxillofacial surgery demonstrate the richness of the research field. Ranging from retrospective studies to case series and technical notes, several study methods have been used to analyze the use of new technologies in our field of interest.

Our Special Issue provides a platform to explore the latest advancements and future trends in these critical areas of our specialty.

In conclusion, the field of oral and maxillofacial surgery stands at the cusp of a new era—an era defined by digitalization, personalized care, and innovation.

This Special Issue serves as a guide to the ingenuity and dedication of the global maxillofacial surgery community, as we strive to push the boundaries of knowledge and redefine the standards of patient care. As we embark on this journey of discovery and advancement, let us remain steadfast in our commitment to excellence and compassion, guided by the principle of putting our patients’ needs first. Together, we will continue to shape the future of oral and maxillofacial surgery, ensuring brighter tomorrows for generations to come.
